# 3-dose of RBD vaccine is sufficient to elicit a long-lasting memory response against SARS-CoV-2 infection

**DOI:** 10.1038/s41392-022-00937-9

**Published:** 2022-03-14

**Authors:** Mengqing Cong, Yunru Yang, Haiyang Tong, Ajmeri Sultana Shimu, Baolong Wang, Qing Li, Fengyin Li, Yi Yang, Tengchuan Jin, Bofeng Li

**Affiliations:** 1 Department of Medical Oncology, The First Affiliated Hospital of USTC, Division of Life Sciences and Medicine, University of Science and Technology of China, Hefei, 230001 China; 2 High Magnetic Field Laboratory, Hefei Institutes of Physical Science, Chinese Academy of Sciences, Hefei, 230031 China; 3 Zhuoyi Biotech Co. Ltd, Changchun, 130616 China

**Keywords:** Infectious diseases, Infectious diseases

**Dear Editor,**

The spread of severe acute respiratory syndrome coronavirus 2 (SARS-CoV-2) led to a global pandemic with 260 million infected people. A variety of vaccines, including the mRNA and recombinant vaccines, were developed to prevent the spread of the disease.^1,2^ There is an urgent need for testing the vaccine-induced long-term memory response based on the immunological principles and identifying the optimal combination of dose and injection manner to provide important information for clinical application.

To date, there is a lack of comprehensive studies to explore the immunological differences of recombinant RBD protein vaccine in immunization doses and schedules. Herein, we systematically compared the immune responses of recombinant RBD protein vaccine immunizing for two doses, three doses, and four doses with 0.5 μg or 5 μg RBD protein in C57BL/6 mice, adjuvanted with aluminum hydroxide and the TLR9 agonists CpG oligodeoxynucleotides (Supplementary Fig. S1a). We titrated serum samples collected from mice at 2 weeks after the last administration. 5RCA (RBD+CpG+Al(OH)_3_) treatment elicited a significant elevation of level and titers of RBD specific IgG than those of 0.5RCA treatment (Fig. 1a and Supplementary Fig. S1b). Notably, these antigen-specific IgGs were IgG1, but not IgG2b (Supplementary Fig. S1c, d). We also detected the inhibition of the binding between RBD and ACE2 by neutralizing antibodies in sera, the IC_50_ titers of 3-dose and 4-dose 5RCA treatment group were significantly higher compared to PBS treated control group, while no significant change in other groups (Supplementary Fig. S1e). To further examine whether a long-lasting memory response was induced by different dose of vaccination, memory response related population,^3^ including germinal center (GC) B cells, T follicular helper (T_FH_) cells, plasmablast cells, plasma cells, and memory B cells were gated and analyzed by flow cytometry (Supplementary Fig. S2). In spleen, a significant augmented percentage and number of (B220^+^IgD^−^GL7^+^Fas^+^) GC B cells and T_FH_ cells (CD4^+^CD44^+^PD-1^+^CXCR5^+^) in 3-dose and 4-dose treated groups (both 0.5 and 5RCA) were observed, but not in 2-dose treated groups (Fig. 1b, c and Supplementary Fig. S3a–d). By using fluorescence-labeled RBD to stain RBD-specific GC B cells in the spleen, we found 3-dose and 4-dose vaccination induced large frequencies and numbers of RBD-specific GC B cells compared with 2-dose injection (Supplementary Fig. S3e, f). Also, long-lived plasma cells migrate into the bone marrow and contribute to antibodies production.^4^ In bone marrow, 3-dose and 4-dose treatments led to a dramatic increase in their frequencies and numbers plasmablast cells (B220^+^CD138^+^), plasma cells (B220^−^CD138^+^), and RBD specific memory B cells (B220^+^IgD^−^GL7^−^CD38^+^) (Fig. 1d–f and Supplementary Fig. S4a–e). The same trend was displayed in the spleen (Supplementary Fig. S5a–h). For T cells activation, we also found remarkable augmented frequencies of CD4^+^CD69^+^ and CD8^+^CD69^+^ T cells in spleen, LN, and lung in the 3-dose and 4-dose RCA treatment group (Supplementary Fig. S6a–f).

**Fig. 1:**
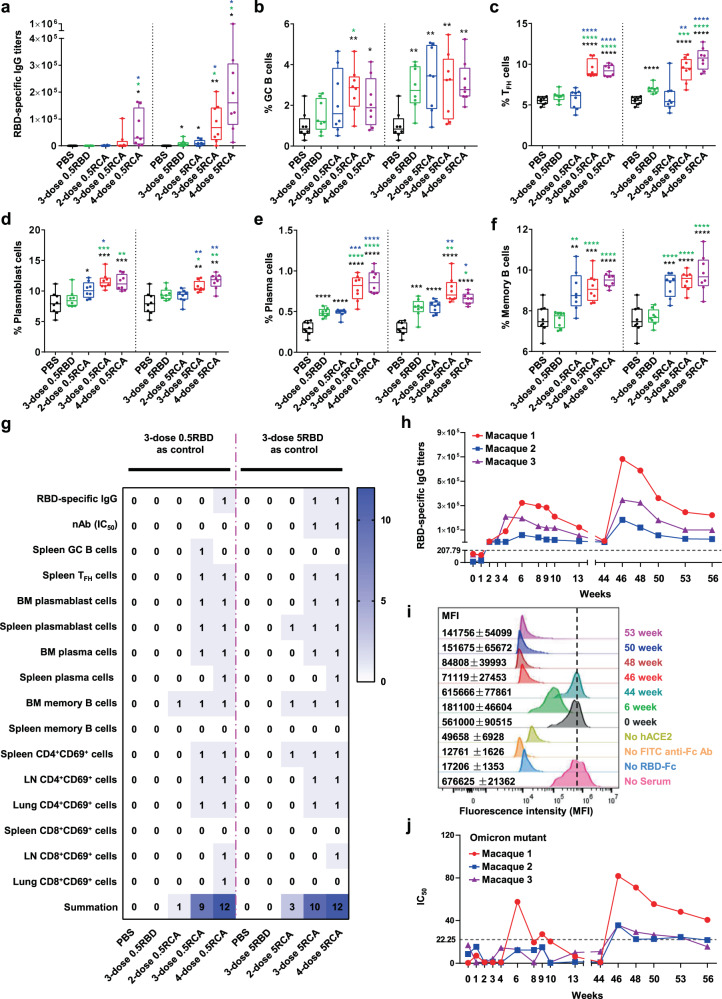
3-dose of RBD vaccine effectively boosts long-term memory response. **a**–**g** C57BL/6 mice were immunized i.p. with 0.5 μg or 5 μg SARS-CoV-2 recombinant RBD protein (321-591aa) adjuvanted with or without 0.25 mg aluminum hydroxide and 10 μg CpG for 2, 3, or 4 times. Blood, spleen, inguinal LNs, bone marrow, and lung were analyzed 14 days after the last dose immunization. 0.5, 0.5 μg RBD; 5, 5 μg RBD; R, RBD; C, CpG; A, aluminum hydroxide. **a** SARS-CoV-2 RBD-specific IgG titers were assessed by endpoint dilution ELISA in mice. **b**, **c** Frequency of murine GC B cells (B220^+^IgD^−^GL7^+^Fas^+^) and T_FH_ cells (CXCR5^+^PD-1^+^) in the spleen. **d**–**f** Frequency of murine plasmablast cells (B220^+^CD138^+^), plasma cells (B220^−^CD138^+^), and memory B cells (GL7^−^CD38^+^) in the bone marrow. Significance was determined by One-way ANOVA or unpaired t-tests. Black (PBS), green (3-dose RBD), blue (2-dose RCA) and red (3-dose RCA) colored asterisks indicated color representative group as a comparison to other groups if *P < 0.05, **P < 0.01, ***P < 0.001, ****P < 0.0001. All data are representative of at least two independent experiments. **g** Summary of significances on different immunization manner in mice. Using 3-dose 0.5RBD or 5RBD as control, counted any significant change (including *, **, and ***) as 1 and add up to a total score. **h**–**j** Three macaques were immunized i.m. with 12.5 μg SARS-CoV-2 recombinant RBD protein adjuvanted with 0.5 mg aluminum hydroxide and 0.5 mg CpG at 0, 1, and 4 weeks for 3 doses. After 40 weeks apart from the third immunization, three macaques were given a fourth immunization with 50 μg SARS-CoV-2 recombinant RBD protein adjuvanted with 0.5 mg aluminum hydroxide and 0.5 mg CpG. Bloods were collected and analyzed at week 0, 1, 2, 3, 4, 6, 8, 9, 10, 13, 44, 46, 48, 50, 53, and 56. **h** SARS-CoV-2 RBD-specific IgG titers were assessed by endpoint dilution ELISA in macaques. The dotted line indicated the cut off value (2.1 × the mean titers of the negative control). **i** The capacity of serum antibodies to inhibit the interaction of RBD and human ACE2. **j** The capacity of serum antibodies to inhibit the interaction of Omicron mutant protein and human ACE2 was assessed by using inhibition ELISA. The dotted line indicated the cut off value (mean of negative control + 2 × SD)

To compare the elicited immune response by different doses of vaccines, we summarized all above data. We collected 16 indicators in spleen, LN, bone marrow, and lung shown in Fig. 1g. We choose the 3-dose RBD as comparisons and input 1 when any significant change (including *, **, and ***) displayed, we calculated tallied scores for 1 and 3 for low (0.5RCA) and high (5RCA) 2-dose administration, while all 3-dose and 4-dose administration got tallied score more than 9. All these comparisons showed greatly boosted immune responses were induced by at least 3 times vaccination. Interestingly, our data showed the 4-dose injection failed to show significant improvement than 3-dose injection, suggesting a consecutive 3-dose administration strategy is sufficient to promote humoral and cellular immune responses.

To further assess whether the protective effect of RCA in non-human primates, 3 macaques were administrated with 3-dose RCA (12.5 µg/macaque) at week 0, 1, 4, with a fourth RCA rechallenge at 44 weeks (Supplementary Fig. S7a). We tracked RBD-specific IgG titers until 56 weeks. As shown in Fig. 1h, it started to increase at week 3 and peaked at week 6. At the time point of week 44, there were almost none detectable IgG for all macaques (9394, 538, and 1836). 2 weeks (week 46) after the 4th vaccination, the RBD specific IgG titer reach a higher peak compared with the first peak at week 6 for individual macaque (683330 vs 323111, 183927 vs 57703, 346240 vs 194628), and even another 10 weeks went by, the IgG titers still kept at high levels (221757, 25163, and 99760). We also examined the IC_50_, which indicated the inhibition effect of macaques’ serum antibody in blocking the interaction between RBD and ACE2. We found a similar pattern for each macaque after 4th immunization (Supplementary Fig. S7b). To further confirm these results, we transfected human ACE2 into HEK-293T cell and used flow cytometry to examine the inhibition effect on the interaction between RBD and hACE2. These hACE2-293T cells were co-incubated with gradient dilution serum collected from immunized mice to compete with added RBD-Fc protein. In this way, a lower MFI (mean fluorescence intensity) indicated serum neutralizing antibody inhibited more chances of RBD and ACE2 binding. We found the MFI of week 6 when macaques received 3 times of vaccination (181100 ± 46604) was greatly lower than MFI of week 0 (561000 ± 90515) and with a restored peak at week 44 (615666 ± 77861). At week 46, 4-dose immunization successfully recalled humoral response with MFI of 71119 ± 27453 and kept the inhibition effect until week 53 (MFI = 141756 ± 54099) (Fig. 1i and Supplementary Fig. S7c). We also examined the kinetics of the existed memory B cells (CD19^+^CD20^+^CD27^+^) in the peripheral blood, 4th vaccination boosted 3.4 fold memory B cells at week 46–56 than week 44 (Supplementary Fig. S7d, e). To determine the neutralization effect of recombinant vaccine on most popular SARS-CoV-2 variants,^5^ including Alpha (B.1.1.7), Beta (B.1.351), Gamma (P.1), Delta (B.1.617.2), and Omicron (B.1.1.529) variants,^6^ we collected the sera from individual macaque between week 0 and 56 from non-human primates. Similar to the kinetic of RBD-specific IgG titers and their IC_50_, a fourth vaccine rechallenge 280 days after the 3rd dose, successfully recalled the memory response and greatly increased neutralization antibodies to bind with RBD mutations (α, β, γ, and δ) (Supplementary Fig. S8a–d) as well as partial inhibition on Omicron mutation (Fig. 1j). Of note, there were no significant changes in the percentage of B cells, CD4^+^ T cells, and CD8^+^ T cells, and serum IL-2, IL-4, IL-5, IL-6, TNF-α, as well as IFN-γ concentrations were kept at the same levels compared to week 0 (Supplementary Fig. S9a–i). These results suggested a good safety profile of our RCA vaccine in non-human primates and supported further clinical vaccine development.

In conclusion, we demonstrated 3-dose vaccination was sufficient to induce high quality humoral immune and long-lasting memory response in mice and non-human primates. It provided fundamental data for 3-dose vaccination to prevent the spread of SARS-CoV-2 virus and its variants based on the immunological principles.

## Supplementary information

supplementary data

## Data Availability

The online version of this article contains supplementary material, which is available to authorized users.
