# Supplementary material for: 3-dose of RBD vaccine is sufficient to elicit a long-lasting memory response against SARS-CoV-2 infection
Source: Signal Transduct Target Ther. 2022 Mar 14;7:84. doi: 10.1038/s41392-022-00937-9 (PMC8918896; doi:10.1038/s41392-022-00937-9)
Supplement: Supplementary file 1 — supplementary data [file 41392_2022_937_MOESM1_ESM.docx]

Supplementary Materials for

**3-Dose of RBD Vaccine is Sufficient to Elicit A Long-lasting**

**Memory Response Against SARS-CoV-2 Infection**

Mengqing cong^1, +^, Yunru Yang^1, +^, Haiyang Tong^2^, Ajmeri Sultana Shimu^1^, Baolong Wang^1^, Qing Li^1^, Fengyin Li^1^, Yi Yang^3^, Tengchuan Jin^1,*^, Bofeng Li^1,*^

^1^ Department of Medical Oncology, The First Affiliated Hospital of USTC, Division of Life Sciences and Medicine, University of Science and Technology of China, Hefei, 230001, China.

^+^ These authors contributed equally: Mengqing cong and Yunru Yang.

^*^Correspondence: Tengchuan Jin, email: jint@ustc.edu.cn, and Bofeng Li, email: [libf@ustc.edu.cn](mailto:libf@ustc.edu.cn).

**This file includes:**

**Materials and Methods**

**Supplementary Fig. S1 to S9**

**Supplementary Fig. S1**. Elevated IgG titers and nAbs are driven by SARS-CoV-2 subunit vaccine immunization in mice.

**Supplementary Fig. S2**. Representative gating strategy for immune cells.

**Supplementary Fig. S3**. 3-dose subunit vaccine induces robust germinal center response in mice.

**Supplementary Fig. S4**. Elevated PC and MBC after vaccination in bone marrow of mice.

**Supplementary Fig. S5**. Elevated PC and MBC after vaccination in spleen of mice.

**Supplementary Fig. S6**. Three doses vaccination induces robust T cells activation in mice.

**Supplementary Fig. S7**. Elevated nAbs and MBC are driven by SARS-CoV-2 subunit vaccine immunization in macaques.

**Supplementary Fig. S8**. Elevated nAbs against SARS-CoV-2 α, β, γ, and δ mutant proteins in macaques.

**Supplementary Fig. S9**. Safety evaluation of SARS-CoV-2 subunit vaccine in macaques.

**Materials and Methods**

***Mice and macaques***

6-8 weeks C57BL/6 male mice were purchased from Shanghai SLAC Laboratory and housed in SPF environment of the University of Science and Technology of China. All animal studies follow the Ethics Committee of the University of Science and Technology of China with approved number USTCACUC202501028.

Three rhesus macaques (Macaca mulatta) were used in this study, listed as macaque 1, macaque 2, and macaque 3. In detail, macaque 1 is female at 3 years old with 3.8 kg body weight; macaque 2 is male at 12 years old with 9 kg body weight; macaque 3 is male at 12 years old with 8.6 kg body weight. Three macaques were immunized in separate cages at the Animal Center of Hefei Institutes of Physical Science Chinese Academy of Sciences and were fed commercial pellets feed, fruits and drinking water. All procedures were conducted under the ethical treatment principles of USTC with approved number 202006220919000464981.

***SARS-CoV-2 RBD and RBD mutants production***

The purification of SARS-CoV-2 RBD was performed as previously described^1^. In brief, we constructed the RBD sequence (321-591aa) into PTT5-TEV-Fc, transiently transfected the expression plasmid into HEK293F cells, and collected the cell supernatant after three days. After loading the cell supernatant on a Protein A column, the target protein was eluted with 0.1 M acetic acid. 5 mM DTT, 1 mM EDTA, and TEV protease were added to digest the RBD-TEV-Fc. After dialysis in 1 × PBS overnight, the sample was loaded onto Protein A plus Ni-NTA columns and the target protein was collected from flow through. SARS-CoV-2 RBD protein was concentrated and stored on a -80°C freezer. RBD mutants (RBD-Alpha (N501Y, A570D), RBD-Beta (K417N, E484K, N501Y), RBD-Gamma (K417T, E484K, N501Y), RBD-Delta (L452R, T478K)) were obtained by PCR site-directed mutagenesis and constructed into the PTT5-TEV-Fc. RBD mutants were purified by same method described above^1^. SARS-CoV-2 B.1.1.529 (Omicron) spike RBD protein (His Tag) was purchased from Sino Biological (40592-V08H121).

***Mice immunization and samples collection***

The vaccine component includes 0.5 μg or 5 μg of recombinant SARS-CoV-2 RBD protein (321-591aa) as immunogen, 0.25 mg aluminum hydroxide and 10 μg CpG as adjuvants. The vaccine volume was adjusted to 0.5 mL with 1 × PBS per mice and injected into the naïve mice i.p.. From day 14 to 107 after the last dose immunization, mice were anesthetized with isoptobarbital. Blood samples were collected and immediately centrifuged at 4000 × g for 15 minutes at 4°C. Serum samples were stored at - 80°C for ELISA and ACE2-RBD inhibition assays. At day 14 after last dose immunization, spleen, LN, bone marrow, and lung were harvested. Spleen and LN were homogenized and filtered through a 70 μm strainer to acquire a single cell suspension. Red blood cells (RBCs) of spleens were lysed using erythrocyte lysate (Beyotime, C3702) for 5-10 minutes on ice. Bone marrows were flushed out of the marrow cavity. Lungs were cut into small pieces and put into 5 mL tubes and digested with 1 mg/mL Collagenase IV (Sigma, C5138-5G) for 1 hour at 37°C with 100 rpm shaking. Cells from all above organs were resuspended in cold 1 × PBS and immediately counted and stained.

***Macaques immunization and samples collection***

Three macaques were immunized i.m. with 12.5 μg recombinant SARS-CoV-2 RBD protein adjuvant with 0.5 mg aluminum hydroxide and 0.5 mg CpG for 3 times at 0, 1 and 4 week. 40 weeks later, a fourth booster immunization (50 μg RBD + 0.5 mg aluminum hydroxide + 0.5 mg CpG) was administrated to the same site as the primary immunization. At 0, 1, 2, 3, 4, 6, 8, 9, 10, 13, 44, 46, 48, 50, 53, and 56 week post-immunization, macaques were anesthetized with isoptobarbital and blood samples were collected to isolate peripheral blood lymphocyte cells (PBMC) and sera or plasma. Sera or plasma were stored at -80°C for ELISA assay, ACE2-RBD inhibition assays.

***Staining and flow cytometry***

Single cell suspensions of spleens, LNs, BM and lungs were blocked with anti-CD16/CD32 monoclonal antibody (Biolegend, 93) for 20 minutes on ice prior to staining. Recombinant RBD protein was conjugated with biotin following product manual (ThermoFisher Scientific, A35358). For staining antigen-specific GC B cells, memory B cells, plasmablast cells and plasma cells, cells were incubated with RBD-biotin for 1 hour, washed and then stained with anti-mouse CD45 (Biolegend, 30-F11), anti-mouse B220 (Biolegend, RA3-6B2), anti-mouse IgD (Biolegend, 11-26c.2a), anti-mouse GL7 (Biolegend, GL7), anti-mouse CD38 (Biolegend, 90), anti-mouse CD138 (Biolegend, 281-2), anti-mouse Fas (Biolegend, SA367H8) and anti-biotin PE (Biolegend, 1D4-C5) antibodies, together with Fixable Viability Dye (BD, 564406). T cells were stained with anti-mouse CD45 (Biolegend, 30-F11), anti-mouse CD4 (Biolegend, GK1.5), anti-mouse CD44 (Biolegend, IM7), anti-mouse PD-1 (Biolegend, 29F.1A12), anti-mouse CXCR5 (Biolegend, L138D7), anti-mouse CD69 (BD, H1.2F3) and anti-mouse CD8α (Biolegend, 53-6.7) antibodies. PBMCs of macaques were stained with anti-NHP CD45 (BD, D058-1283), anti-human CD19 (Biolegend, HIB19), anti-human CD20 (Biolegend, 2H7), anti-human CD27 (Biolegend, O323), anti-human/NHP CD3 (BD, SP34-2), anti-human CD4 (Biolegend, OKT4), anti-human CD8a (Biolegend, RPA-T8). Cells were stained on ice for 30 minutes, washed and then resuspended in cold 1 × PBS and analyzed by flow cytometry.

***ELISA and competitive ELISA***

The purified RBD (0.2 μg/well) was coated on Nunc MaxiSorp plates at 4°C overnight. Washed the plate with 1 × PBS three times and blocked by 5% no-fat milk at room temperature for 2 hours. 1:100 as an initial dilution ratio and a 1:3 serial dilution of the sera were added to the plates at room temperature for 1 hour and then washed three times by 1 × PBST (1 × PBS with 0.1% Tween-20). 1:10000 HRP-goat-anti-mouse IgG (Sangon Biotech, D110087) was added to the plates and incubated for 1 hour at room temperature with three times wash thereafter. TMB solution (Beyotime, P0209) was added to the plates and incubated in dark for 7 minutes. Stopped the reaction by 1 M H_2_SO_4_ and the absorbance at 450 nm was measured. The cut off value for positivity was set at 2.1 × the OD value of the negative control^2, 3^. An arbitrary value of 1 was assigned to the samples with OD values below the cut off value.

Antibodies elicited by infection or vaccination can block the RBD and ACE2 receptor interaction. Based on that, a competitive ELISA was designed to evaluate the neutralization activity of serum samples from different vaccination strategies^4^. Briefly, 0.3 μg/well RBD or RBD mutants (RBD-Alpha, RBD-Beta, RBD-Gamma, RBD-Delta) were added to the plates together with 1:2-1:5 as an initial dilution ratio and a 1:3 serial dilution of the sera. Then 10 nM biotin-hACE2-Fc. and 1:5000 HRP-streptavidin (Beyotime, A0303) were added to each well. The rest of the experimental procedures were same as the ELISA assay. The IC_50_ was calculated by fitting the inhibition (Inhibition = (1 - OD value of Sample / OD value of positive Control) × 100%) from serially diluted serum to a sigmoidal dose-response curve. The cut off value for positivity was set at mean of negative control (serum obtained from macaques before the start of the experiment) + 2 × SD (standard deviation)^5, 6^.

***Cell competitive binding experiment***

According to previously report^7^, we constructed the full length of human ACE2 into the PTT5 expression plasmid (without Fc) and transfected it to HEK293T cells by polyethyleneimine (Polyscience), 36 hours later collected HEK293T cells. 1:5 as an initial dilution ratio and a 1:2 serial dilution of the sera and 5 μg/ml RBD-Fc were added to a 96-well plate containing transfected cells and incubated at room temperature for 1 hour. After 2 times washing, FITC labeled goat anti-human IgG1 (H+L) (Beyotime, A0556) was added to the plates and incubated for 1 hour at room temperature. Cells were resuspended in FACS buffer for flow cytometry.

***Statistical analysis***

All statistical analyses were carried out by GraphPad Prism version 7.04. Significance of differences among groups was conducted using unpaired One-way ANOVA or unpaired t-tests. * p < 0.05, **p < 0.01, ***p < 0.001 and ****p < 0.0001 was used to set statistical significance.

**REFERENCES**

1. Ma, H. et al. Potent Neutralization of SARS-CoV-2 by Hetero-bivalent Alpaca Nanobodies Targeting the Spike Receptor-Binding Domain. J Virol (2021).

2. Wu, Z. et al. Safety, tolerability, and immunogenicity of an inactivated SARS-CoV-2 vaccine (CoronaVac) in healthy adults aged 60 years and older: a randomised, double-blind, placebo-controlled, phase 1/2 clinical trial. Lancet Infect Dis 21, 803-812 (2021).

3. van Doremalen, N. et al. ChAdOx1 nCoV-19 vaccine prevents SARS-CoV-2 pneumonia in rhesus macaques. Nature 586, 578-582 (2020).

4. Zeng, W. et al. Characterization of SARS-CoV-2-specific antibodies in COVID-19 patients reveals highly potent neutralizing IgA. Signal Transduct Target Ther 6, 35 (2021).

5. Sokal, A. et al. Maturation and persistence of the anti-SARS-CoV-2 memory B cell response. Cell 184, 1201-1213 e1214 (2021).

6. Jorrissen, P. et al. Antibody Response to SARS-CoV-2 Membrane Protein in Patients of the Acute and Convalescent Phase of COVID-19. Front Immunol 12, 679841 (2021).

7. Tai, W. et al. A novel receptor-binding domain (RBD)-based mRNA vaccine against SARS-CoV-2. Cell Res 30, 932-935 (2020).

**
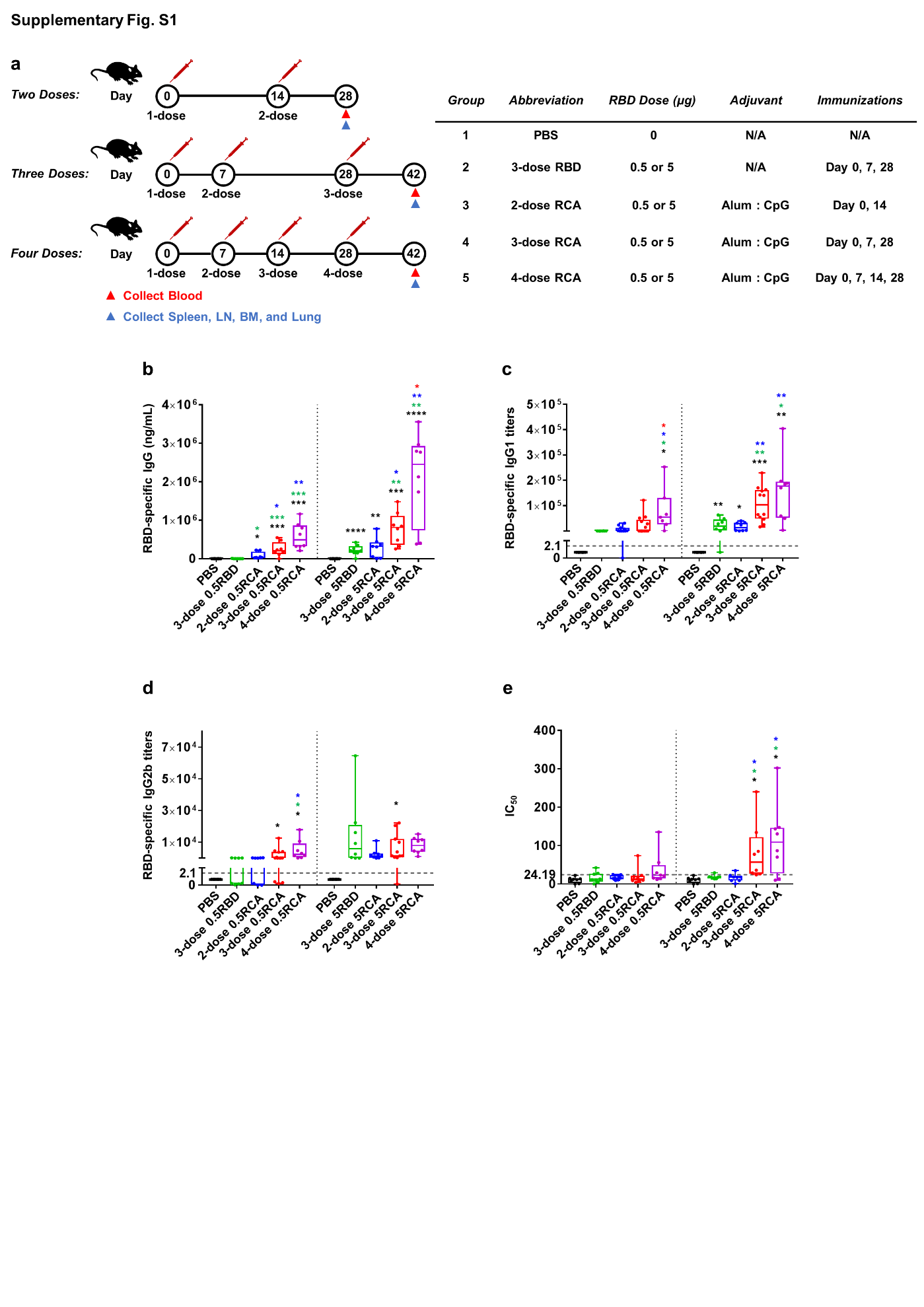
Supplementary Fig. S1.**

**Supplementary Fig. S1. Elevated IgG titers and nAbs are driven by SARS-CoV-2 subunit vaccine immunization in mice.** (a) Schematic diagrams of 2-dose, 3-dose, and 4-dose immunization. C57BL/6 mice were immunized i.p. with 0.5 μg or 5 μg SARS-CoV-2 recombinant RBD protein (321-591aa) adjuvanted with or without 0.25 mg aluminum hydroxide and 10 μg CpG for 2, 3, or 4 times. Blood, spleen, inguinal LNs, bone marrow and lung were analyzed 14 days after the last dose immunization. R, RBD; C, CpG; A, aluminum hydroxide. (b) SARS-CoV-2 RBD-specific IgG levels. (c-d) SARS-CoV-2 RBD-specific IgG1 and IgG2b titers. The dotted line indicates the cut off value for positivity (2.1), which was set at 2.1 × the mean titers of the negative control. (e) The capacity of serum antibodies to inhibit the interaction of RBD and human ACE2 were assessed by ACE2-RBD inhibition ELISA. The dotted line indicates the cut off value for positivity (24.19), which was set at mean of negative control + 2 × SD. Data are representative of two independent experiments with n ≥ 8 per group. Black (PBS), green (3-dose RBD), blue (2-dose RCA) and red (3-dose RCA) colored asterisks indicated color representative group as a comparison to other groups if *P < 0.05, **P < 0.01, ***P < 0.001, ****P < 0.0001.

**
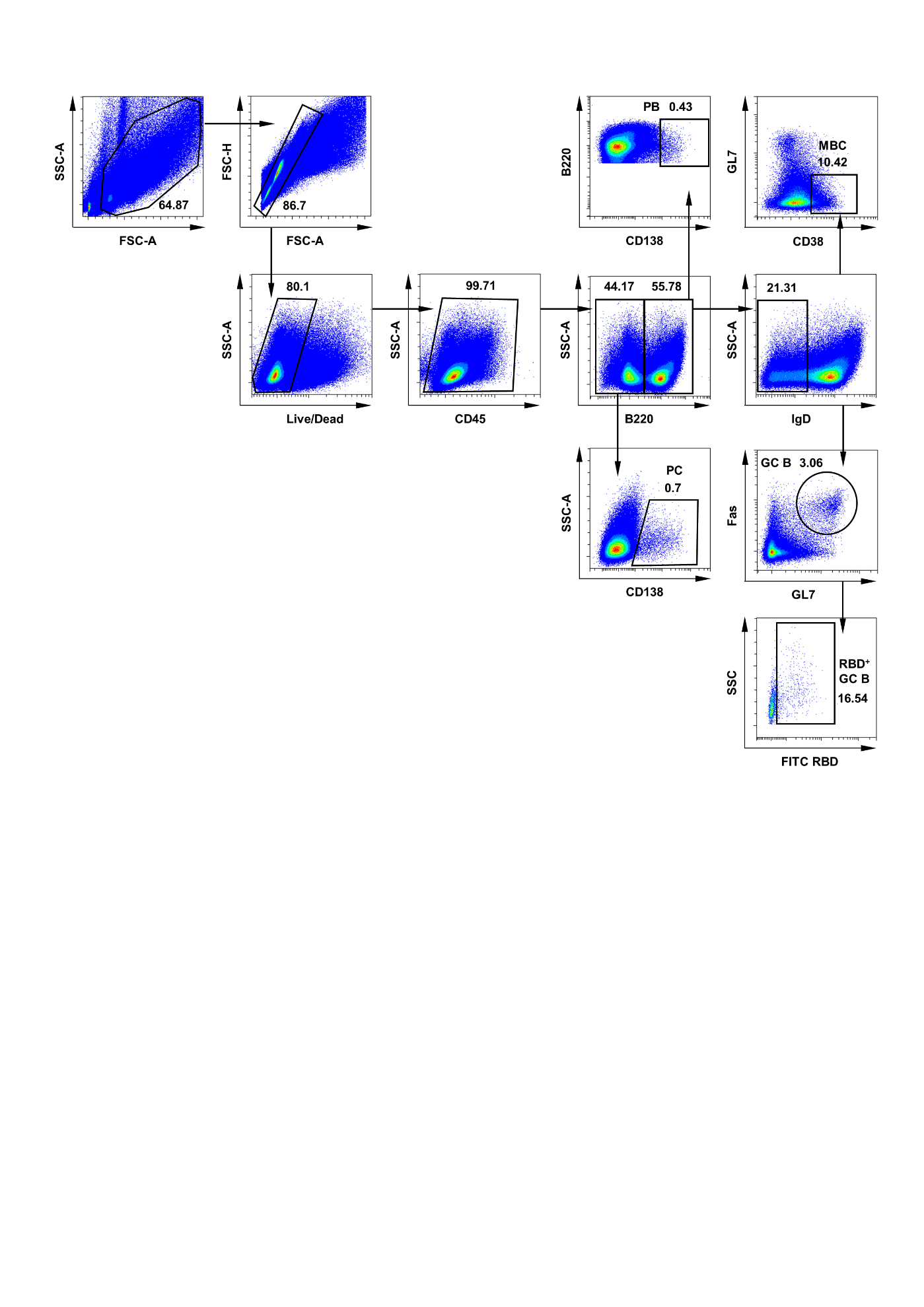
 Supplementary Fig. S2.**

**Supplementary Fig. S2. Representative gating strategy for immune cells.** Gated GC B cells, plasmablast cells, plasma cells, and memory B cells were analyzed by flow cytometry and CytExpert 2.4 software.

**
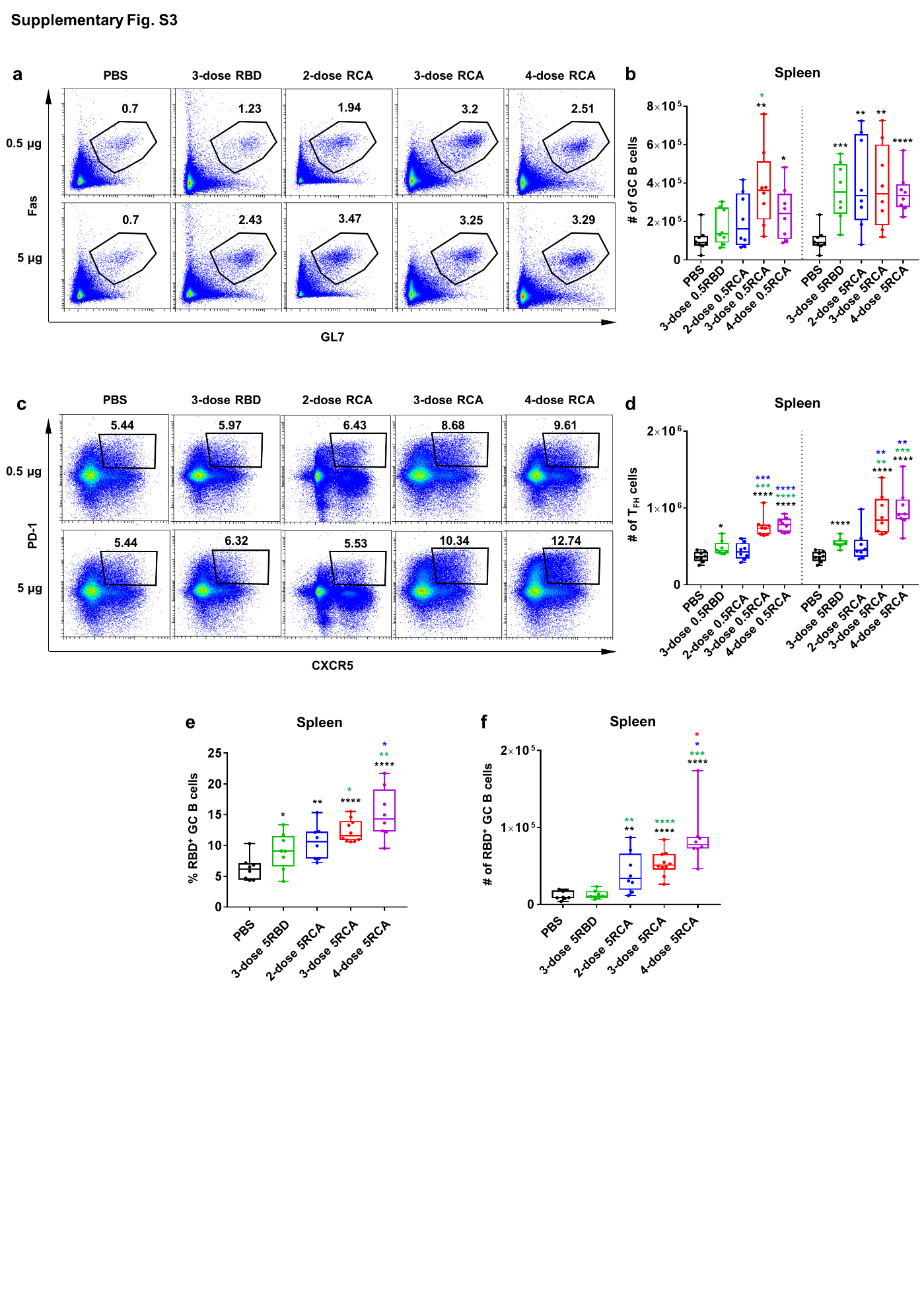
Supplementary Fig. S3.**

**Supplementary Fig. S3. 3-dose subunit vaccine induces robust germinal center response in mice.** (a) Representative flow cytometry dot plots showing GC B cells. (b) Numbers of GC B cells in spleen. (c) Representative flow cytometry dot plots showing T_FH_ cells. (d) Numbers of T_FH_ cells in spleen. (e-f) Frequency (e) and absolute counts (f) of RBD^+^ GC B cells (RBD^+^GL7^+^Fas^+^) in spleen. Data are representative of two independent experiments with n ≥ 8 per group. Black (PBS), green (3-dose RBD), blue (2-dose RCA) and red (3-dose RCA) colored asterisks indicated color representative group as a comparison to other groups if *P < 0.05, **P < 0.01, ***P < 0.001, ****P < 0.0001.

**
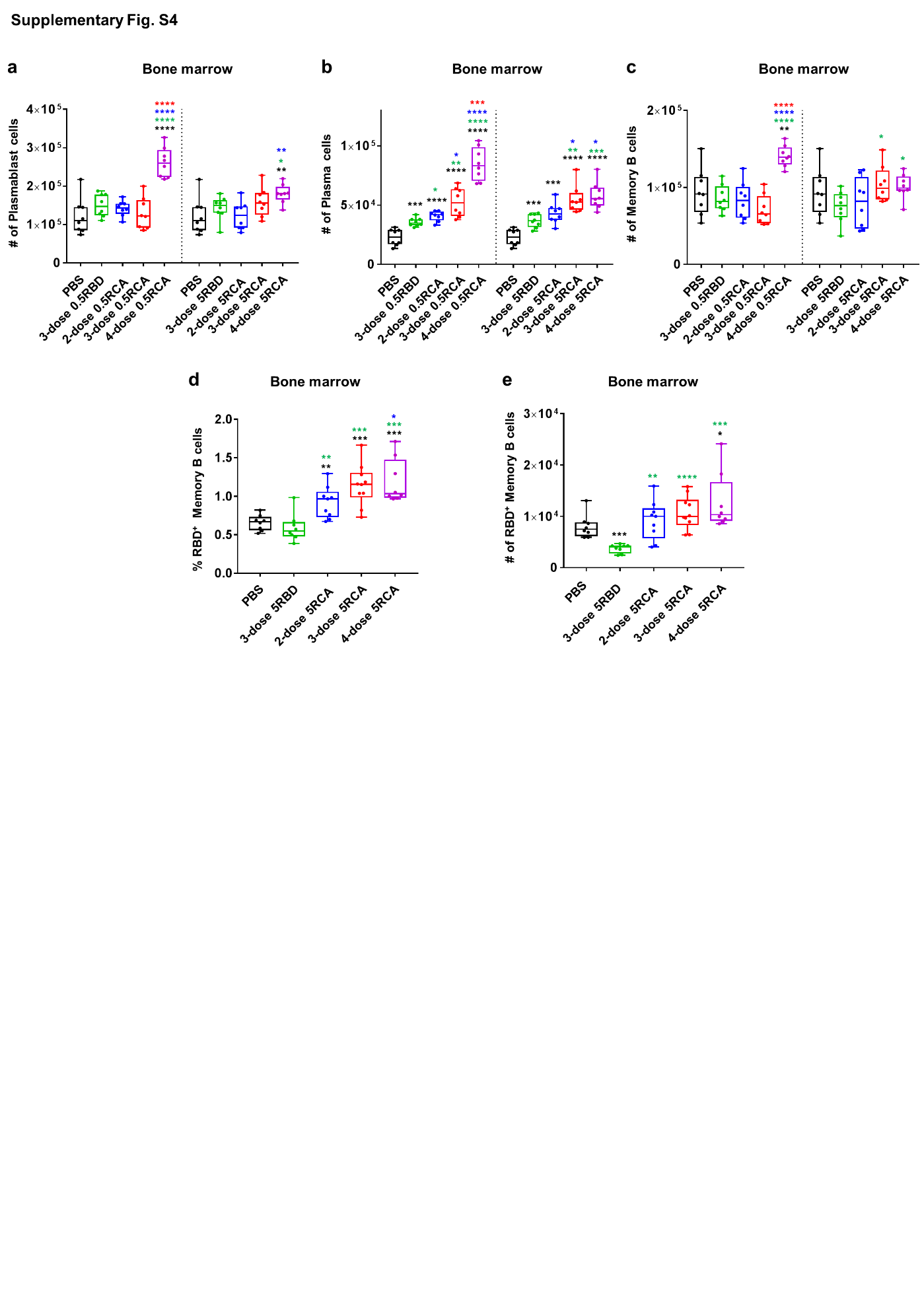
Supplementary Fig. S4.**

**Supplementary Fig. S4. Elevated PC and MBC after vaccination in bone marrow of mice.** (a) Number of plasmablast cells (B220^+^CD138^+^) in bone marrow. (b) Number of plasma cells (B220^-^CD138^+^) in bone marrow. (c) Number of memory B cells (GL7^-^CD38^+^) in bone marrow. (d-e) Frequency (d) and absolute counts (e) of RBD^+^ memory B cells (RBD^+^GL7^-^CD38^+^) in bone marrow. Data are representative of two independent experiments with n ≥ 8 per group. Black (PBS), green (3-dose RBD), blue (2-dose RCA) and red (3-dose RCA) colored asterisks indicated color representative group as a comparison to other groups if *P < 0.05, **P < 0.01, ***P < 0.001, ****P < 0.0001.

**
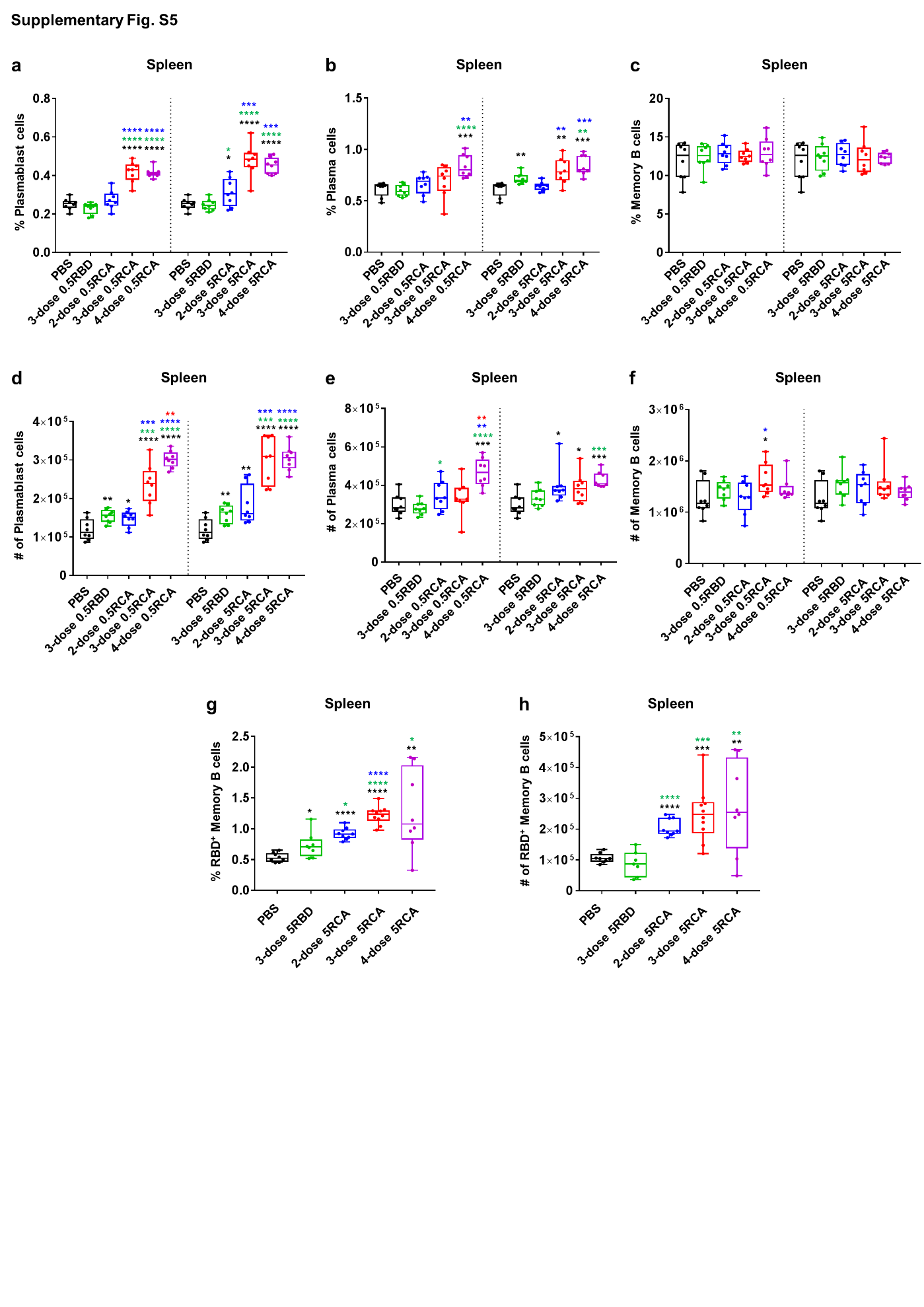
Supplementary Fig. S5.**

**Supplementary Fig. S5. Elevated PC and MBC after vaccination in spleen of mice.** (a and d) Frequency (a) and absolute counts (d) of plasmablast cells (B220^+^CD138^+^) in spleen. (b and e) Frequency (b) and absolute counts (e) of plasma cells (B220^-^CD138^+^) in spleen. (c and f) Frequency (c) and absolute counts (f) of memory B cells (GL7^-^CD38^+^) in spleen. (g-h) Frequency (g) and absolute counts (h) of RBD^+^ memory B cells (RBD^+^GL7^-^CD38^+^) in spleen. Data are representative of two independent experiments with n ≥ 8 per group. Black (PBS), green (3-dose RBD), blue (2-dose RCA) and red (3-dose RCA) colored asterisks indicated color representative group as a comparison to other groups if *P < 0.05, **P < 0.01, ***P < 0.001, ****P < 0.0001.

**
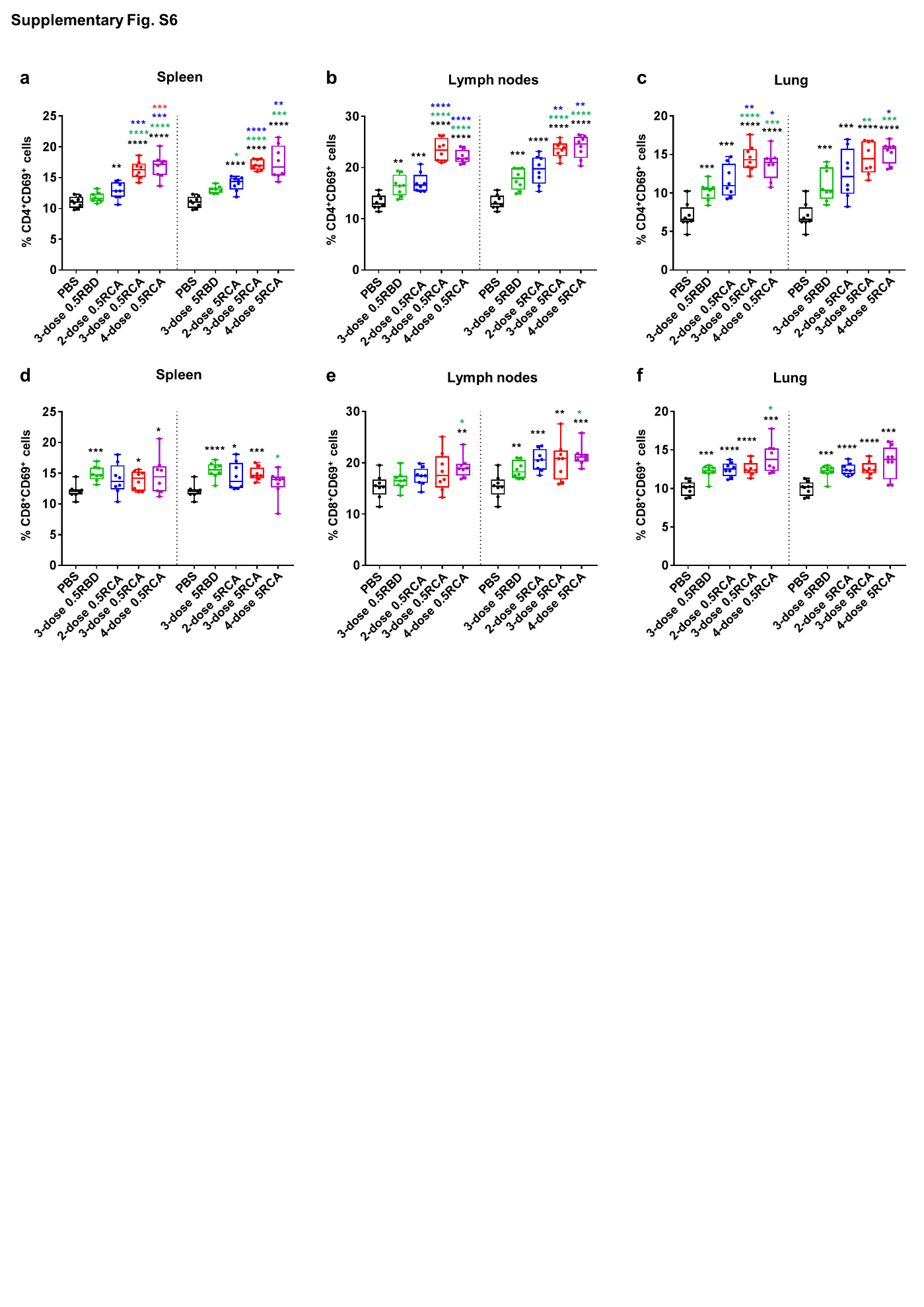
 Supplementary Fig. S6.**

**Supplementary Fig. S6. Three doses vaccination induces robust T cells activation in mice.** (a-f) Frequency of CD4^+^CD69^+^ cells and CD8^+^CD69^+^ cells in spleen (a and d), inguinal LNs (b and e), and lung (c and f). Data are representative of two independent experiments with n ≥ 8 per group. Black (PBS), green (3-dose RBD), blue (2-dose RCA) and red (3-dose RCA) colored asterisks indicated color representative group as a comparison to other groups if *P < 0.05, **P < 0.01, ***P < 0.001, ****P < 0.0001.

**
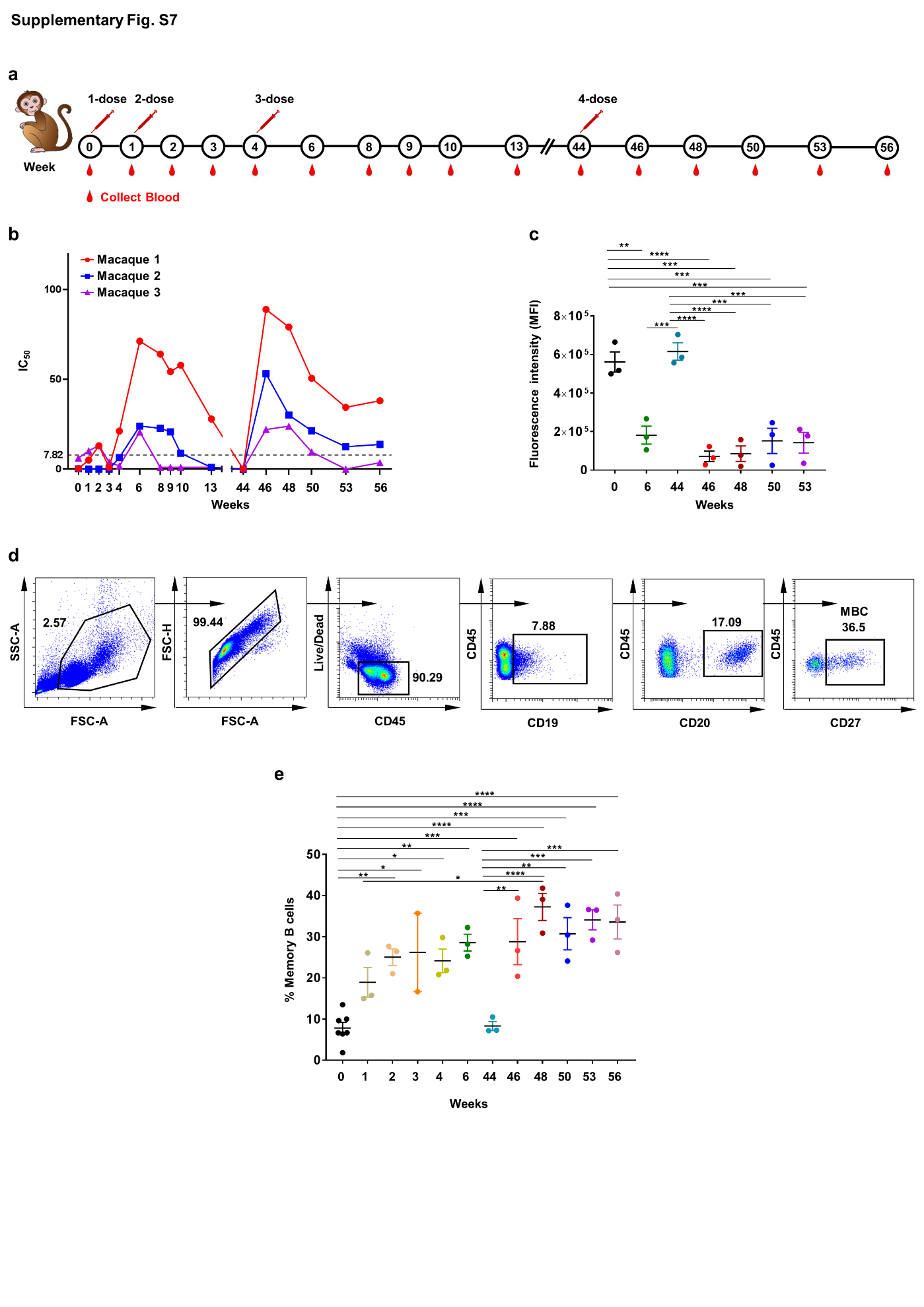
Supplementary Fig. S7.**

**Supplementary Fig. S7. Elevated nAbs and MBC are driven by SARS-CoV-2 subunit vaccine immunization in macaques.** Three macaques were immunized i.m. with 12.5 μg SARS-CoV-2 recombinant RBD protein adjuvanted with 0.5 mg aluminum hydroxide and 0.5 mg CpG at 0, 1, and 4 weeks for 3 doses. After 40 weeks apart from the third immunization, three macaques were given a fourth immunization with 50 μg SARS-CoV-2 recombinant RBD protein adjuvanted with 0.5 mg aluminum hydroxide and 0.5 mg CpG. Bloods were collected and analyzed at week 0, 1, 2, 3, 4, 6, 8, 9, 10, 13, 44, 46, 48, 50, 53 and 56. (a) Schematic diagram of 3-dose immunization with fourth rechallenge. (b) The capacity of macaques’ serum antibodies to inhibit the interaction of RBD and human ACE2 were assessed by ACE2-RBD inhibition ELISA. The dotted line indicates the cut off value for positivity (7.82), which was set at mean of negative control + 2 × SD. (c) The capacity of serum antibodies to inhibit the interaction of RBD and human ACE2 was further confirmed by cell competitive binding experiment detection. FITC mean fluorescence intensity (MFI) of indicated time points was shown by the statistical analysis. (d) Representative flow cytometry dot plots showing memory B cells. (e) Frequency of memory B cells (CD45^+^CD19^+^ CD20^+^CD27^+^) in blood. Symbols represent individual macaque. Data were graphed as mean ± SEM. One-way ANOVA or unpaired t-tests were conducted according to the distribution of the data. *P < 0.05, **P < 0.01, ***P < 0.001, ****P < 0.0001.

**
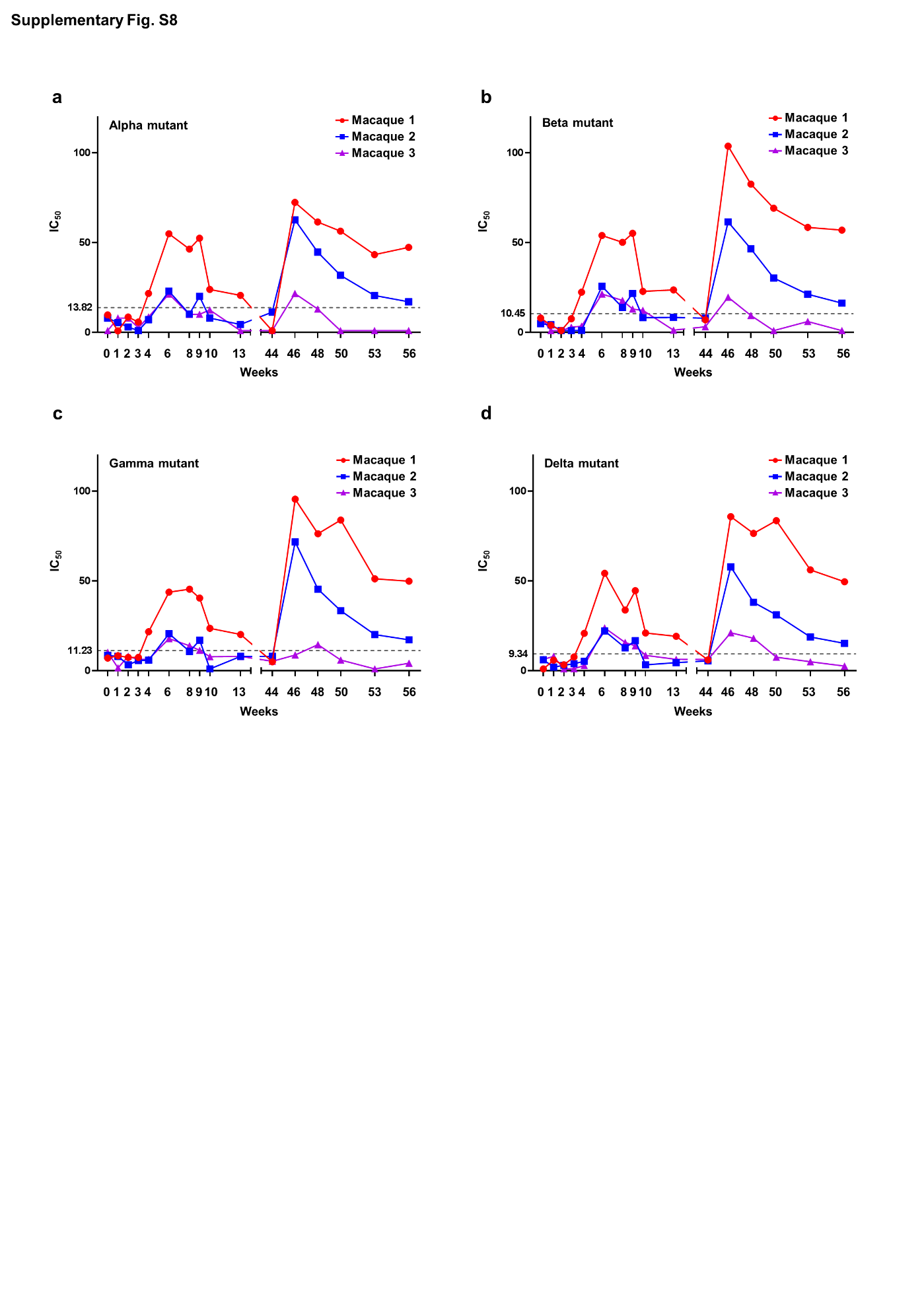
Supplementary Fig. S8.**

**Supplementary Fig. S8. Elevated nAbs against SARS-CoV-2 α, β, γ, and δ mutant proteins in macaques.** (a-d) The capacity of serum antibodies to inhibit the interaction of α (a), β (b), γ (c) or δ (d) mutant proteins and human ACE2 were assessed using ACE2-RBD inhibition ELISA in macaques. The cut off value of α (a), β (b), γ (c), or δ (d) mutants for positivity was set at 13.82, 10.45, 11.23, and 9.34, respectively, which represented mean of negative control + 2 × SD. Three macaques were analyzed. Symbols represent individual macaque.


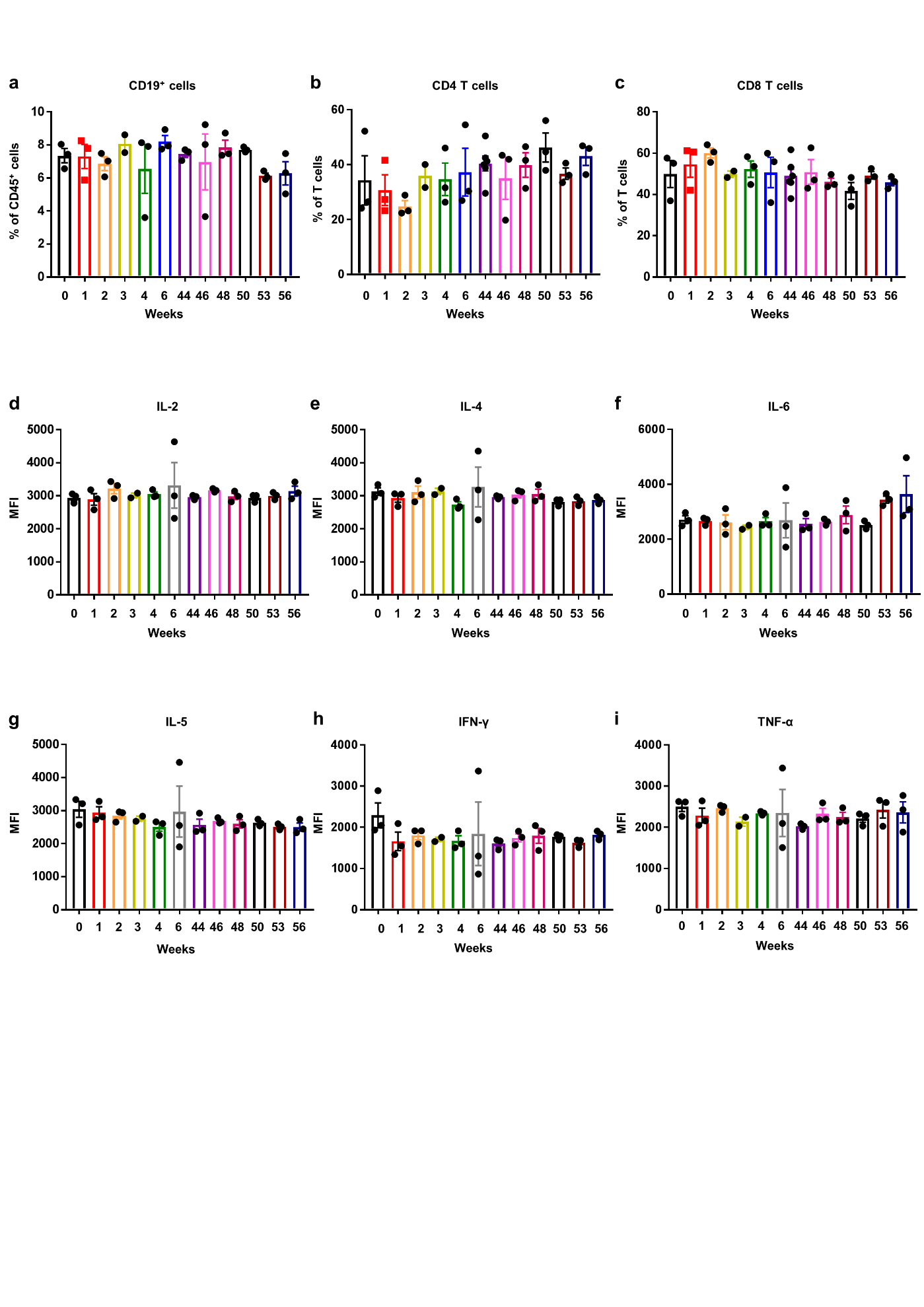
**Supplementary Fig. S9.**

**Supplementary Fig. S9. Safety evaluation of SARS-CoV-2 subunit vaccine in macaques.** (a-c) Frequencies of CD19^+^ cells (a), CD4^+^ T cells (b), and CD8^+^ T cells (c) from macaques' peripheral blood collected at week 0, 1, 2, 3, 4, 6, 44, 46, 48, 50, 53, and 56. (d-i) Mean fluorescence intensity (MFI) of Th1/Th2 cytokines were assessed using BD^TM^ Cytometric Bead Array (CBA) Non-Human Primate Th1/Th2 Cytokine Kit for macaques’ sera at week 0, 1, 2, 3, 4, 6, 44, 46, 48, 50, 53 and 56. Th1/Th2 cytokines include IL-2 (d), IL-4 (e), IL-6 (f), IL-5 (g), IFN-γ (h) and TNF-α (i). In (a)-(i), n = 3 macaques were analyzed. Symbols represent individual macaque. Data were graphed as mean ± SEM. One-way ANOVA or unpaired t-tests were conducted according to the distribution of the data.
